# Treatment of hormone positive uterine leiomyosarcoma with aromatase inhibitors

**DOI:** 10.1186/2045-3329-4-5

**Published:** 2014-06-26

**Authors:** Eirini Thanopoulou, Khin Thway, Komel Khabra, Ian Judson

**Affiliations:** 1 Sarcoma Unit, Royal Marsden NHS Foundation Trust, Chelsea, London SW3 6JJ, UK

**Keywords:** Uterine leiomyosarcoma, Aromatase inhibitors, Hormonal treatment

## Abstract

**Background:**

Aromatase inhibitors (AIs) have not been used consistently as part of the management of hormone receptor positive uterine leiomyosarcomas (ULMS). As a result, the published data regarding the efficacy of AIs in this subtype of ULMS are sparse.

**Methods:**

We performed a retrospective electronic medical record review of patients with ULMS treated with an AI, in the 1st or the 2nd line setting, at the Sarcoma Unit of the Royal Marsden Hospital between 2001 and 2012. We assessed progression-free survival (PFS), objective response and toxicities and explored the correlation of the intensity of the hormone receptor status, as well as of the grade with PFS.

**Results:**

Sixteen patients with measurable advanced ULMS were treated with an AI in our unit. All of them were oestrogen receptor (ER) and progesterone receptor (PgR) positive. Letrozole was used in all patients as 1st line endocrine therapy, while exemestane was mainly prescribed as 2nd line (83%). Median PFS in 1st line was 14 months (95% CI: 0 – 30 months), and prolonged PFS was more likely to be observed in patients with low grade compared to high grade ULMS (20 months vs. 11 months), and in moderately/strongly ER positive compared to weakly ER positive ULMS (20 months vs. 12 months). Best response was partial response (PR) in 2/16 patients (12.5%) and clinical benefit (CB), defined as complete response (CR) + PR + stable disease ≥6 months, was observed in 10/16 patients (CB rate (CBR) 62.5%). Median duration of 2nd line was 3 months and median PFS was not reached. The 1-year progression-free rate for the 2nd line AI was 80%. Best response was PR in one patient and CBR was 50%. AIs were well tolerated in both lines of treatment.

**Conclusions:**

In this population of patients with hormone positive ULMS, AIs achieved a significant CBR (62.5%) in 1st line, which was retained in 2nd line (CBR: 50%). The relatively prolonged median PFS (14 months), along with the favourable toxicity profile could place AIs among the first choices of systemic treatment in hormone positive ULMS, preferably in strongly positive (>90%), and/ or low grade and low volume disease.

## Background

Uterine leiomyosarcomas (ULMS) account for 1–2% of all uterine malignancies [1]. They exhibit an aggressive natural history, with recurrence rates of 50-70% and an overall 5-year survival of less than 50% in early stages and less than 15% in advanced stages [2]. The mainstay of treatment of localized ULMS comprises total abdominal hysterectomy (TAH), bilateral salpingo-oophorectomy (BSO) and excision of all resectable tumours [2]. In the absence of established adjuvant treatment, depending on the histopathological report (i.e. surgical margins, size, grade etc.) adjuvant chemotherapy, radiotherapy or combined treatment are sometimes offered [3-5]. For women with advanced, unresectable ULMS, chemotherapy is given with palliative intent; however, the median duration of response is limited to 6–8 months [6-8]. Therapeutic options are limited for patients who progress following standard chemotherapeutic regimens, although recently the multitargeted tyrosine kinase inhibitor pazopanib has been approved for this indication [9]. Thus, there is an urgent need to identify new active treatments.

Gynaecological sarcomas exhibit a variable rate of oestrogen receptor (ER) and progesterone receptor (PgR) expression [1]. In ULMS, ER has been reported to be positive in 25–60% of cases and PgR in 35–60% respectively [10-13]. Aromatase inhibitors (AIs) have been introduced in the treatment of ULMS [1]. The main mechanism of action is inhibition of aromatase activity in peripheral adipose tissue, resulting in profound reduction in circulating oestrogen levels [1]. AIs may also inhibit directly the aromatase activity in tumour tissue [12].

Few data are available about hormone-positive ULMS; mainly with case reports [14], small retrospective studies [15,16] and recently one prospective single-arm phase II clinical trial [17]. According to this trial (27 patients), letrozole met the protocol definition of active agent in metastatic ULMS that was ER and/or PgR positive [17]. The benefit, in terms of prolongation of PFS, was significant in patients with strongly (>90%) ER and PR tumours [17]. This observation, in line with previous retrospective studies [16], suggested that oestrogen manipulation possibly has an active role in disease control of this subtype of ULMS [17].

AIs have a favourable toxicity profile with the majority of side effects being mild and attributed to the oestrogen deprivation they induce [1,16,17]. They are administered at the same dosages as in breast cancer treatment [1]. With this in mind, we sought to record our single institution’s experience in treating ULMS patients with AIs*.*

## Methods

We performed a retrospective study of patients with ULMS treated with an AI at the Sarcoma Unit of the Royal Marsden Hospital (RMH) from January 2001 to July 2012. Patients were identified using the prospective Sarcoma Unit database and confirmed by pharmacy records. Patients were excluded if they had received an AI as treatment for breast cancer or received concomitant chemotherapy. Patients’ electronic medical records were reviewed for age at diagnosis, stage, sites of metastases, volume of metastatic disease, tumour grade, hormone receptor status (ER and PgR), performance status, prior treatments, type and dose of AI used and toxicities. In addition, we recorded the presence or absence of co-morbidities.

All patients had surgical biopsies reviewed by the RMH Department of Pathology (two dedicated soft tissue pathologists), which confirmed the diagnosis of ULMS and tumour grade. Currently, there is no formally validated grading system for ULMS. In the absence of one, the French Federation of Cancer Centers Sarcoma Group (FNCLCC) system has been used in RMH, as for other sarcomas [18]. In some centres, the Bell criteria for uterine smooth muscle tumours (or Stanford USMT criteria) are used [16,19]. When these criteria are strictly applied, ULMS are defined as intrinsically high grade, although low grade ULMS may still exist [20].

Immunohistochemistry for ER and PgR was performed on formalin fixed, paraffin embedded, representative, whole sections of tumour. Deparaffinized tumour sections were stained for ER and PgR (both supplied prediluted from Ventana Systems UK Ltd, Salisbury, UK) using heat-induced epitope retrieval. Appropriate positive and negative controls were used throughout. ER and PgR status was determined semi-quantitatively and assigned as ‘weak’, ‘moderate’ or ‘strong’ in tumour nuclei.

The primary end-point of the study was PFS for the patients with advanced disease, and disease-free survival (DFS) for the adjuvant patients, defined in both cases as time from the start of AI treatment until disease progression or death from any cause, with censoring of patients who were lost or had reached neither endpoint at date of last follow-up. The Kaplan–Meier method was used to estimate PFS. Objective response rate (ORR), i.e. the rate of CR and PR, and clinical benefit rate (CBR), i.e. the rate of CR, PR, and SD for at least 6 months were evaluated by Response Evaluation Criteria in Solid Tumours (revised RECIST guideline, version 1.1) criteria [21]. The tumour assessments were performed with computerised tomography (CT) every 2 months for the first 12 months and then every 3 months thereafter. We explored the correlation of ER/PgR status and tumour grade with objective response and PFS. Toxicity was graded using the National Cancer Institute Common Terminology Criteria for Adverse Events (CTCAE) version 4.02.

The study was approved by the Committee for Clinical Research of RMH.

## Results

### Patient and tumour characteristics

We identified 29 patients with ULMS treated with an AI from January 2001 to July 2012. We excluded 8 cases that were treated in another institution and 5 patients that did not have evidence of measurable disease at the time of AI initiation. The demographics and tumour characteristics of the remaining 16 patients with advanced measurable disease are listed in Table 1 and their tumour characteristics are listed in Table 2. The median age at time of AI initiation as 1st line hormonal treatment was 55 years (range 39–72). Fourteen patients (87.5%) were postmenopausal at the time of treatment.

**Table 1:** Patient characteristics in the 1st line endocrine setting (n = 16)

**Variable**	**n (%), median (range)**
**Median age at AI initiation**	55 years (39–72)
**Performance status**	
0	4 (25%)
1	10 (62.5%)
2	1 (6.25%)
3	1 (6.25%)
**Menopausal status**	
Premenopausal	2 (12.5%)
Postmenopausal	14 (87.5%)
**Median body mass index**	27.6 kg/m2 (20.3–52.7)
**Number of co-morbidities**	
0–1	13 (81%)
2–3	3 (19%)
4–5	0 (0%)
**Stage at diagnosis**	
Non metastatic	11 (69%)
Locally recurrent/ metastatic	5 (31%)
**Sites of metastases at time of AI initiation**	
Lung	12 (75%)
[Lung as only site of metastases]	10 (62.5%)
Pelvis	4 (25%)
Peritoneum	3 (19%)
**Tumour volume at time of AI initiation**	
Low	6 (37.5%)
High	10 (62.5%)
**Number of metastases**	
Oligometastatic	7 (44%)
Multiple	9 (56%)

AI, aromatase inhibitor.

**Table 2:** Tumour characteristics in the 1st line endocrine setting (n = 16)

**Variable**	**n = 16 (%)**
**Histological grade (Stanford)**	
Low	8 (50%)
High	8 (50%)
**Histological grade (FNCLCC)**	
Low	7 (44%)
Intermediate	3 (19%)
High	6 (37%)
**Hormone receptor status**	
**ER**	
Moderate to strong (grade 2–3)	13 (81%)
Weak (grade 1)	3 (19%)
**PgR**	
Moderate to strong (grade 2–3)	10 (62.5%)
Weak (grade 1)	6 (37.5%)
**ER and PgR**	
Moderate to strong (grade 2–3)	10 (62.5%)
Weak (grade 1)	2 (12.5%)
**% ER staining**	
>90%	10 (62.5%)
>50%	14 (87.5%)
10-50%	1 (6.25%)
Unknown	1 (6.25%)
**% PgR staining**	
>90%	6 (37.5%)
>50%	11 (68.75%)
10-50%	1 (6.25%)
0%	2 (12.5%)
unknown	2 (12.5%)

ER, oestrogen receptor; PgR, progesterone receptor.

Tumours were classified according to FNCLCC criteria, as low-grade ULMS in 7 patients (44%), intermediate grade in 3 (19%) patients and high-grade ULMS in 6 patients (44%). We also classified our patients according to Stanford USMT criteria; 8 patients were found to have high-grade ULMS (50%) and 8 patients low-grade ULMS (50%; Table 2). Due to the small number of our patients, we eventually analysed them according to Stanford USMT criteria.

ER status was determined semi-quantitatively in all (n = 16) patients, and assigned as ‘weak’, ‘moderate’ or ‘strong’ in tumour nuclei. Three of these 16 patients (19%) had weak (1+) ER positive staining, while 13 patients (81%) had moderate to strong (2-3+) ER positive staining. With regards to PgR status: 10 (62.5%) had a moderate to strong PgR staining, and 6 (37.5%) had a weak PgR staining. Of the 10 patients with moderate to strong PgR positive disease, all were also moderately to strongly ER positive (Table 2). Moreover, in Table 2 is depicted the classification of tumours by the extent of ER/PgR as reported by the percentage of tumour expressing each receptor [17].

Prior surgical and systemic treatment details are provided in Table 3. All of the 16 evaluable patients had measurable disease at time of AI treatment. Six (37.5%) patients had low volume (defined as the absence of any tumour deposit >2 cm in longest diameter on radiographic imaging) measurable disease [16]. However, 10 (62.5%) had multiple metastases, with only one of these having low volume disease. Sites of disease included lung in 12 patients (75%), pelvis in 4 (25%) and peritoneum in 3 (19%).

**Table 3:** Patient treatment details (n = 16)

**Variable - 1st line**	**n (%), median (range)**
**Initial management at diagnosis of ULMS**	
Surgical resection alone	3 (19%)
Surgical resection and BSO	13 (81%)
**Number of prior chemotherapy regimens**	
0	12 (75%)
1	2 (12.5%)
2	2 (12.5%)
**Prior exogenous estrogens**	
Tamoxifen	3 (19%) , 60 months
HRT	4 (25%), 48 months (24–96)
**Prior pelvic radiotherapy**	1 (6%)
**Median interval between diagnosis (surgery) and letrozole initiation**	37 months (2–308)

ULMS, uterine leiomyosarcoma; BSO, bilateral salpingo-oophorectomy; HRT, hormonal replacement therapy.

Four patients (25%) received hormonal replacement therapy (HRT) with a median duration of treatment of 48 months (24–96; Table 3). Three patients progressed whilst on HRT; withdrawal of HRT led to regression of disease (equivalent to partial response with hormonal manipulation) in two patients that lasted for 10 months and 84 months respectively, while the other patient started treatment with letrozole. Three (19%) of the 16 patients received tamoxifen (Table 3); two of them were diagnosed with early breast cancer prior to their diagnosis of ULMS (and one of them also received HRT), while the third received tamoxifen as a therapeutic manoeuvre for ULMS and progressed one month later.

### Aromatase inhibitors as 1st line endocrine therapy

Letrozole was prescribed as 1st line therapy (2.5 mg daily) in all 16 patients (Table 1). Goserelin, a gonadotropin-releasing hormone agonist, was administered to suppress ovarian function in the two premenopausal women of our cohort, in order to permit treatment with an AI. One patient switched from letrozole to anastrozole due to side effects and eventually stopped treatment due to poor tolerance. Twelve (75%) patients received AI as 1st line treatment without receiving any anticancer agent prior to AI. Two patients had received gemcitabine plus docetaxel prior to 1st line AI, with one of them starting letrozole as maintenance treatment. The other two patients received two lines of chemotherapy prior to starting 1st line letrozole.

### Aromatase inhibitors as 2nd line endocrine treatment

From these 16 patients, 6 of them received 2nd line endocrine treatment with exemestane (25 mg; 5 patients) or anastrozole (1 mg; 1 patient), when their disease progressed. The demographics and tumour characteristics of those 6 patients are listed in Table 4. The median age at time of 2nd line AI initiation was 56 years (range 40–74). One patient was premenopausal; hence goserelin was prescribed to suppress ovarian function. Five out of 6 tumours were classified as low-grade ULMS. With regards to ER and PgR status, five patients had moderate to strong (grade 2–3) ER with the four of them having also grade 2–3 PgR staining. Moreover, five patients had very high expression of ER (>90%; Table 4). All patients had measurable disease at time of 2nd line AI treatment; 4 patients had progressed in the lung and the other 2 patients to the pelvis and peritoneum (Table 4).

**Table 4:** Patient and tumour characteristics in the 2nd line endocrine setting (n = 6)

**Variable -2nd line**	**n (%), median (range)**
**Median age at AI initiation**	56 years (40–74)
**Performance status**	
0	2 (33%)
1	4 (67%)
**Menopausal status**	
Premenopausal	1 (17%)
Postmenopausal	5 (83%)
**Number of co-morbidities**	
0-1	5 (83%)
2-3	1 (17%)
**Sites of metastases at time of AI initiation**	
Lung	4 (67%)
[Lung as only site of metastases]	4 (67%)
Peritoneum/pelvis	2 (33%)
**Tumour volume at time of AI initiation**	
Low	2 (33%)
High	4 (67%)
**Histological grade (Stanford)**	
Low	5 (83%)
High	1 (17%)
**Hormone receptor status**	
**ER**	
Moderate to strong (grade 2–3)	5 (83%)
Weak (grade 1)	1 (17%)
**PgR**	
Moderate to strong (grade 2–3)	4 (67%)
Weak (grade 1)/ NA	2 (33%)
**ER and PgR**	
Moderate to strong (grade 2–3)	4 (67%)
Weak (grade 1)	1 (17%)
**% ER staining**	
>90%	5 (83%)
unknown	1 (17%)
**% PgR staining**	
>90%	2 (33%)
>50%	3 (50%)
0%	1 (17%)
unknown	2 (33%)

AI, aromatase inhibitor; ER, oestrogen receptor; PgR, progesterone receptor.

### Objective responses to 1st line with aromatase inhibitor

The median duration of 1st line treatment with letrozole was 13 months (range 2–29). No patient achieved a CR, while PR was observed in 2 (12.5%). The first patient had low-grade ULMS strongly ER and PgR positive with low volume peritoneal disease. The other had high grade, moderately ER and PgR positive, oligometastatic disease and received letrozole as maintenance treatment after completing 6 cycles of chemotherapy, which may make difficult to assess accurately the therapeutic impact of letrozole alone. The duration of response in these patients was 10 and 11 months respectively.

Ten patients (62.5%) had SD as best response, with 8 of them maintaining it for more than 6 months. The duration of stable disease (median PFS) was 20.5 months (95% CI: 8.7 – 32.2 months), with 8 patients stopping letrozole because of disease progression, one because of poor tolerance, while one is still on letrozole (27+ months). Six of these patients had low-grade ULMS according to both grading systems and the remaining four had high-grade disease. ER status for all except one patient with SD was moderate to strong, while 7 patients were strongly PgR positive, 2 PgR negative and 1 patient had unknown PgR status. The CBR (CR + PR + SD >6 months) was 62.5% (10/16 patients).

Best response was progression of disease (PD) in 4 patients (25%). Three patients had high-grade disease; 2 patients had weak staining for ER and PgR, while the third strongly expressed both receptors. The fourth patient had low grade ULMS and ER and PgR strongly positive and she received letrozole after progressing on two previous lines of chemotherapy with symptomatic high volume disease.

### Objective responses to 2nd line with aromatase inhibitor

The median duration of 2nd line AI therapy was 3 months (range 3–22 months). One patient achieved PR (after progression of peritoneal disease) but stopped treatment after 3 months due to toxicity. She had low grade, strongly ER and PgR positive disease.

Three patients had SD as best response and are continuing to date (duration of treatment 2, 6 and 22 months respectively). All of them had high volume disease with multiple lung metastases. Moreover all had low grade ULMS, with two of them expressing strongly both receptors and the third being weakly ER positive with unknown PgR status.

Two patients (on anastrozole and exemestane respectively) had PD as best response (duration of treatment 3 months); one of them with low-grade, strongly ER and PgR positive disease was switched to 3rd line treatment with exemestane, while the other patient had high grade, strongly ER/PgR positive disease and was eventually treated with 1st line chemotherapy.

### Progression-free survival of 1st line with aromatase inhibitor

**Figure 1:**
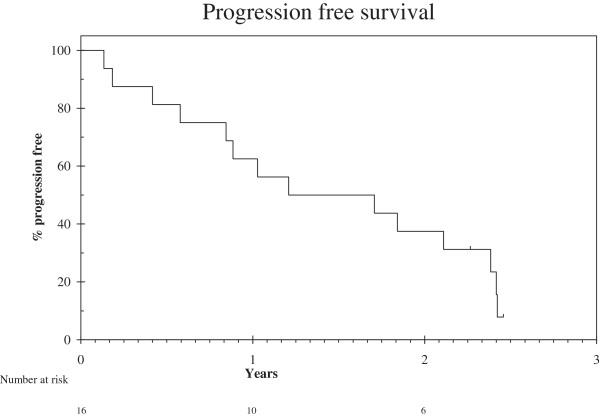
Progression free survival defined as date of start of first line treatment to date of progression or death.

**Figure 2:**
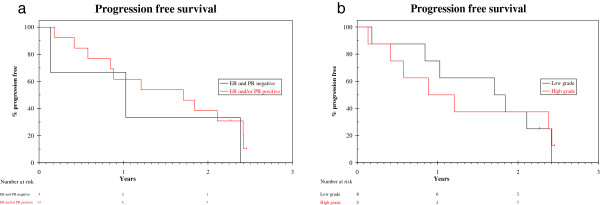
**Progression free survival stratified by hormone receptor status and tumour grade. a**. Progression free survival stratified by hormone receptor status. **b**. Progression free survival stratified by tumour grade.

At last follow-up, 4 (25%) of the 16 patients had died with disease progression, 5 (31%) were alive with disease progression, 2 (12.5%) were alive on 1st line letrozole without progression, 2 (12.5%) patients had stopped letrozole and exemestane respectively due to toxicity and were alive without progression and 3 (19%) patients were alive on 2nd line exemestane without progression (Figure 1). Median PFS for 1st line letrozole was 14 months (95% CI: 0–30 months). The 1-year progression-free rate was 63% (95% CI: 39%-87%).Figure 2 illustrates the difference in PFS in 1st line treatment with letrozole between patients with moderate and strong ER and/or PgR status and those with tumours with weak ER and PgR status. Patients with both hormone receptors moderately to strongly positive had superior median PFS [20 months (95% CI: 7– 34)] compared to those with weak ER and PgR expression [median PFS 12 months (95% CI: 0 – 29)]. However, due to the small number of patients it is not possible to drive safe conclusions due to lack of statistical significance in the overall cohort analysis.Moreover, patients with low-grade ULMS (n = 8) had superior median PFS [20 months (95% CI: 7 – 34)], compared to those with high-grade ULMS [n = 8; median PFS 11 months (95% CI: 0 – 30)], as demonstrated in Figure 2b. Accordingly, the 1-year progression-free rate for the low-grade ULMS was 75% (95% CI: 45% - 100%) compared to 50% (15% - 87%) in the high grade ULMS.

### Progression-free survival of 2nd line with aromatase inhibitor

Median PFS for the 2nd line AI has not been reached after a median follow-up of 6 months. The 1-year progression-free rate for the 2nd line AI was 80% (95% CI: 21% - 97%).

### Toxicities during aromatase inhibitor treatment - any dose reduction/ discontinuation

Toxicities during the 1st line treatment with letrozole were noted among 31% of patients and all were mild (grade 1–2); specifically, hot flushes were recorded in 5 patients (31%), lethargy in 4 (25%), arthralgias/myalgias in 4 (25%), and weight gain in 1 patient (6%). One patient developed osteoporosis and started supplementation treatment. Another patient developed significant nausea and chest tightness and eventually discontinued anastrozole.

During the 2nd line treatment only one patient developed grade 1 arthralgias and another (premenopausal) developed grade 2 hot flushes, headaches and rashes and discontinued treatment. The rate of discontinuation of AIs secondary to toxicity among all patients was low (2/16 patients).

## Discussion

Progestins were among the first hormonal modulators to be explored in the management of metastatic ULMS [1]. However, AIs are currently the preferred hormonal agents for 1st line treatment, because of their high therapeutic index. Moreover, AIs have shown activity as 2nd line treatment in progestin-resistant ULMS [1].

This case series adds the data regarding the role of AIs in the management of patients with hormone positive ULMS [15-17], though the results should be interpreted cautiously, as our study is retrospective and the number of patients is small. In our cohort of 16 patients, with measurable advanced hormone positive ULMS, objective response was observed in 12.5%, and clinical benefit rate in 62.5%; median PFS was 14 months (95% CI: 0 – 30). Moreover, prolonged PFS was more likely to be observed in patients with low grade compared to high grade ULMS [20 months (95% CI: 7 – 34) vs. 11 months (95% CI: 0 – 30)]. Similarly, prolonged PFS was more likely to be observed in patients with moderately to strongly ER and/or PgR ULMS compared to those with weakly hormone receptors’ expression [20 months (95% CI: 7– 34) vs. 12 months (95% CI: 0 – 29)].

To our knowledge, this is the first case series of advanced hormone positive ULMS reporting outcomes of 2nd line endocrine treatment. In our cohort of 6 patients, objective response was observed in 1 patient (PR), who had low grade strongly ER/PgR positive disease (>90% ER expression). Three patients had SD and are continuing to date, the two of them for more than 6 months (CBR = 50%; 3/6 patients). All of them had high volume disease, low grade ULMS, and two of them were strongly ER ER/PgR positive (>90% ER expression). Two patients progressed (strongly ER/PgR positive, high-grade ULMS).

In terms of side effects, a constellation of oestrogen deprivation symptoms were the reason for discontinuation of AIs in 2 out of the 16 patients (12%). Moreover, the detrimental effect of HRT and tamoxifen has also been shown in our study, with withdrawal of HRT leading to disease regression in three patients [1].

In both our and O’Cearbhaill et al. [16] retrospecitve studies, patients with low grade ULMS had superior PFS compared to patients with high grade ULMS; both studies used the Stanford USMT criteria for grade [19], though grade has not been found to be of prognostic significance in ULMS [22]. In the only prospective phase II trial of letrozole as 1st line in patients with advanced ULMS [17] they used mitotic index, which has been shown to have prognostic significance [22,23]. There was no significant association found between mitotic index and PFS [17]. Of note, currently there is no formally validated grading system for ULMS [16,18,19].

In the only prospective trial of George et al. [17], the best response was stable disease in (14/26) of patients. The median PFS was 12 weeks for the whole cohort, but 3 patients with high expression of both ER and PgR (>90%) were free of disease progression after 6 months on study [17]. Moreover, there was a significant association between ER status (>90%) and prolonged PFS, while such association was not confirmed with mitotic index [17]. In the largest retrospective study in 34 patients with advanced ULMS, the best objective response of AIs was partial response in 9% of patients, all of whom were ER positive; the CBR was 41% [16]. The median PFS was 2.9 months, although both objective responses and prolonged PFS were more likely to be observed in patients with ER and/or PgR expression [16].

By adding our findings to the above studies, it can be deducted that there is a correlation between high (>90%) ER and PgR expression and prolonged PFS in 1st line treatment with AIs. The slight differences between the published ORR and PFS of the phase II trial and our data may merely reflect the difference in the percentage of patients that were strongly ER/PgR positive. In the phase II trial, only 11% (3/26) of patients’ tumours expressed ER in >90% of tumour cells [17], while in our cohort, 62.5% (10/16) of patients had tumours that expressed ER in >90% of cells. Such detailed description on the intensity of ER expression of the ULMS tumours was not presented in other retrospective study [16].

The above data may also suggest that the ER and PgR expression may not be only a prognostic factor [10,13], but also a predictive marker in ULMS, although the precise role of hormone receptors in the molecular biology of ULMS remains unclear [17]. The significant prolongation of PFS in patients with very strong expression of ER/PgR, in both our study and the recently published trial [17], suggest that AIs may play an active therapeutic role in this hormone-drive subtype of ULMS, whose ER cut-off status is set to 90%.

## Conclusions

There is clearly a subset of ULMS with a more indolent clinical behaviour, which is clinically characterised by disease-free interval greater than 6 months [24]. This ULMS subset seems to be mostly ER/PgR [13,16] and includes patients that often present with low-volume disease [16]. In line with the published studies [16,17], AIs could be considered active agents for 1st line treatment in hormone positive advanced ULMS, preferably for these highly selected ULMS patients with strongly ER and/or PgR positive status (>90%) and/or low grade and/or low volume disease, as it appears to be more limited in unselected cohorts [16,17]. Moreover, in such highly selected patients with advanced ULMS, it could also be considered 2nd line treatment with exemestane (steroidal AI), in view of the positive outcomes in our small cohort (CBR: 50%). As expected, AIs were well tolerated.

## Abbreviations

AIs: Aromatase inhibitors; BSO: Bilateral salpingo-oophorectomy; CB: Clinical benefit; CBR: Clinical benefit rate (CR + PR + SD ≥ 6 months); CR: Complete response; CTCAE: Common Terminology Criteria for Adverse Events; DFS: disease-free survival; ER: Oestrogen receptor; FNCLCC: Fédération Nationale des Centres de Lutte Contre le Cancer (French Federation of Cancer Centers Sarcoma Group); HRT: Hormonal replacement therapy; ORR: Objective response rate; PR: Partial response; PgR: Progesterone receptor; PD: Progression of disease; PFS: Progression-free survival; RECIST: Response evaluation criteria in solid tumours; RMH: Royal Marsden Hospital; SD: Stable disease; TAH: Total abdominal hysterectomy; ULMS: Uterine leiomyosarcomas; USMT: Uterine smooth muscle tumours.

## Competing interests

The authors declare that they have no competing interests.

## Authors’ contributions

ET and IJ contributed to the conception, design, drafting of the manuscript and coordinated the manuscript drafting. KT performed the histopathological analyses of the tumours. KK performed the statistical analysis. All authors read and approved the final manuscript. All authors read and approved the final manuscript.

## References

[B1] Thanopoulou E Judson I Hormonal therapy in gynecological sarcomas Expert Rev Anticancer Ther 2012 12 885 894 10.1586/era.12.74 22845404

[B2] D’Angelo E Prat J Uterine sarcomas: a review Gynecol Oncol 2010 116 131 139 10.1016/j.ygyno.2009.09.023 19853898

[B3] Reed NS Mangioni C Malmstrom H Scarfone G Poveda A Pecorelli S Tateo S Franchi M Jobsen JJ Coens C Teodorovic I Vergote I Vermorken JB Phase III randomised study to evaluate the role of adjuvant pelvic radiotherapy in the treatment of uterine sarcomas stages I and II: an European Organisation for Research and Treatment of Cancer Gynaecological Cancer Group Study (protocol 55874) Eur J Cancer 2008 44 808 818 10.1016/j.ejca.2008.01.019 18378136

[B4] Omura GA Blessing JA Major F Lifshitz S Ehrlich CE Mangan C Beecham J Park R Silverberg S A randomized clinical trial of adjuvant adriamycin in uterine sarcomas: a Gynecologic Oncology Group Study J Clin Oncol 1985 3 1240 1245 3897471 10.1200/JCO.1985.3.9.1240

[B5] Hensley ML Ishill N Soslow R Larkin J Abu-Rustum N Sabbatini P Konner J Tew W Spriggs D Aghajanian CA Adjuvant gemcitabine plus docetaxel for completely resected stages I-IV high grade uterine leiomyosarcoma: Results of a prospective study Gynecol Oncol 2009 112 563 567 10.1016/j.ygyno.2008.11.027 19135708

[B6] Hensley ML Blessing JA Mannel R Rose PG Fixed-dose rate gemcitabine plus docetaxel as first-line therapy for metastatic uterine leiomyosarcoma: a Gynecologic Oncology Group phase II trial Gynecol Oncol 2008 109 329 334 10.1016/j.ygyno.2008.03.010 18534250 PMC2504727

[B7] Muss HB Bundy B DiSaia PJ Homesley HD Fowler WC Jr Creasman W Yordan E Treatment of recurrent or advanced uterine sarcoma. A randomized trial of doxorubicin versus doxorubicin and cyclophosphamide (a phase III trial of the Gynecologic Oncology Group) Cancer 1985 55 1648 1653 10.1002/1097-0142(19850415)55:8<1648::AID-CNCR2820550806>3.0.CO;2-7 3884128

[B8] Sanfilippo R Grosso F Jones RL Banerjee S Pilotti S D’Incalci M Dei Tos AP Raspagliesi F Judson I Casali PG Trabectedin in advanced uterine leiomyosarcomas: a retrospective case series analysis from two reference centers Gynecol Oncol 2011 123 553 556 10.1016/j.ygyno.2011.08.016 21917307

[B9] van der Graaf WT Blay JY Chawla SP Kim DW Bui-Nguyen B Casali PG Schoffski P Aglietta M Staddon AP Beppu Y Le Cesne A Gelderblom H Judson IR Araki N Ouali M Marreaud S Hodge R Dewji MR Coens C Demetri GD Fletcher CD Dei Tos AP Hohenberger P Pazopanib for metastatic soft-tissue sarcoma (PALETTE): a randomised, double-blind, placebo-controlled phase 3 trial Lancet 2012 379 1879 1886 10.1016/S0140-6736(12)60651-5 22595799

[B10] Akhan SE Yavuz E Tecer A Iyibozkurt CA Topuz S Tuzlali S Bengisu E Berkman S The expression of Ki-67, p53, estrogen and progesterone receptors affecting survival in uterine leiomyosarcomas. A clinicopathologic study Gynecol Oncol 2005 99 36 42 10.1016/j.ygyno.2005.05.019 15992918

[B11] Bodner K Bodner-Adler B Kimberger O Czerwenka K Leodolter S Mayerhofer K Estrogen and progesterone receptor expression in patients with uterine leiomyosarcoma and correlation with different clinicopathological parameters Anticancer Res 2003 23 729 732 12680175

[B12] Kitaoka Y Kitawaki J Koshiba H Inoue S Ishihara H Teramoto M Honjo H Aromatase cytochrome P450 and estrogen and progesterone receptors in uterine sarcomas: correlation with clinical parameters J Steroid Biochem Mol Biol 2004 88 183 189 10.1016/j.jsbmb.2003.11.013 15084350

[B13] Leitao MM Jr Hensley ML Barakat RR Aghajanian C Gardner GJ Jewell EL O’Cearbhaill R Soslow RA Immunohistochemical expression of estrogen and progesterone receptors and outcomes in patients with newly diagnosed uterine leiomyosarcoma Gynecol Oncol 2012 124 558 562 10.1016/j.ygyno.2011.11.009 22085894

[B14] Hardman MP Roman JJ Burnett AF Santin AD Metastatic uterine leiomyosarcoma regression using an aromatase inhibitor Obstet Gynecol 2007 110 518 520 10.1097/01.AOG.0000267533.56546.c2 17666649

[B15] Ioffe YJ Li AJ Walsh CS Karlan BY Leuchter R Forscher C Cass I Hormone receptor expression in uterine sarcomas: prognostic and therapeutic roles Gynecol Oncol 2009 115 466 471 10.1016/j.ygyno.2009.08.014 19767065

[B16] O’Cearbhaill R Zhou Q Iasonos A Soslow RA Leitao MM Aghajanian C Hensley ML Treatment of advanced uterine leiomyosarcoma with aromatase inhibitors Gynecol Oncol 2010 116 424 429 10.1016/j.ygyno.2009.10.064 19932916 PMC4852374

[B17] George S Feng Y Manola J Nucci MR Butrynski JE Morgan JA Ramaiya N Quek R Penson RT Wagner AJ Harmon D Demetri GD Krasner C Phase 2 trial of aromatase inhibition with letrozole in patients with uterine leiomyosarcomas expressing estrogen and/or progesterone receptors Cancer 2014 120 738 743 10.1002/cncr.28476 24222211

[B18] Coindre JM Nguyen BB Bonichon F de Mascarel I Trojani M Histopathologic grading in spindle cell soft tissue sarcomas Cancer 1988 11 2305 9 3365658 10.1002/1097-0142(19880601)61:11<2305::aid-cncr2820611126>3.0.co;2-m

[B19] Bell SW Kempson RL Hendrickson MR Problematic uterine smooth muscle neoplasms. A clinicopathologic study of 213 cases Am J Surg Pathol 1994 18 535 558 10.1097/00000478-199406000-00001 8179071

[B20] Veras E Zivanovic O Jacks L Chiappetta D Hensley M Soslow R “Low-grade leiomyosarcoma” and late-recurring smooth muscle tumors of the uterus: a heterogenous collection of frequently misdiagnosed tumors associated with an overall favorable prognosis relative to conventional uterine leiomyosarcomas Am J Surg Pathol 2011 35 1626 1637 10.1097/PAS.0b013e31822b44d2 21921786

[B21] Eisenhauer EA Therasse P Bogaerts J Schwartz LH Sargent D Ford R Dancey J Arbuck S Gwyther S Mooney M Rubinstein L Shankar L Dodd L Kaplan R Lacombe D Verweij J New response evaluation criteria in solid tumours: revised RECIST guideline (version 1.1) European journal of cancer 2009 45 228 247 10.1016/j.ejca.2008.10.026 19097774

[B22] Pautier P Genestie C Rey A Morice P Roche B Lhomme C Haie-Meder C Duvillard P Analysis of clinicopathologic prognostic factors for 157 uterine sarcomas and evaluation of a grading score validated for soft tissue sarcoma Cancer 2000 88 1425 1431 10.1002/(SICI)1097-0142(20000315)88:6<1425::AID-CNCR21>3.0.CO;2-3 10717626

[B23] D’Angelo E Espinosa I Ali R Gilks CB Rijn M Lee CH Prat J Uterine leiomyosarcomas: tumor size, mitotic index, and biomarkers Ki67, and Bcl-2 identify two groups with different prognosis Gynecol Oncol 2011 121 328 333 10.1016/j.ygyno.2011.01.022 21316747

[B24] Giuntoli RL 2nd Garrett-Mayer E Bristow RE Gostout BS Secondary cytoreduction in the management of recurrent uterine leiomyosarcoma Gynecol Oncol 2007 106 82 88 10.1016/j.ygyno.2007.02.031 17434579

